# Effects of Eicosapentaenoic Acid vs Eicosapentaenoic/Docosahexaenoic Acids on Cardiovascular Mortality: Meta-Analysis of Clinical Trials

**DOI:** 10.1016/j.jacadv.2025.102149

**Published:** 2025-09-19

**Authors:** John P. Sheppard, Leonard Palatnic, Suvasini Lakshmanan, Thomas Drago, Jaspreet Bhogal, Sion K. Roy, Deepak L. Bhatt, Matthew J. Budoff, John R. Nelson

**Affiliations:** a Smidt Heart Institute, Cedars-Sinai Medical Center, Los Angeles, California, USA; b University at Buffalo, Buffalo, New York, USA; c Lundquist Institute/Harbor-UCLA Medical Center, Torrance, California, USA; d Mount Sinai Fuster Heart Hospital, Icahn School of Medicine at Mount Sinai, New York, New York, USA; e California Cardiovascular Institute, Fresno, California, USA

**Keywords:** cardiovascular mortality, docosahexaenoic acid, eicosapentaenoic acid, meta-analysis, omega-3 fatty acids

## Abstract

**Background:**

Purified eicosapentaenoic acid (EPA) and mixed eicosapentaenoic/docosahexaenoic acids (EPA/DHA) are omega-3 polyunsaturated fatty acids (n-3 PUFAs) of interest for preventing cardiovascular disease (CVD) as adjunct to statins. Randomized clinical trial (RCT) evidence continues to emerge, including data from the RESPECT-EPA (Randomized Trial for Evaluation in Secondary Prevention Efficacy of Combination Therapy–Statin and Eicosapentaenoic Acid) trial, but n-3 PUFAs’ roles in prevention remains controversial.

**Objectives:**

The objective of the study was to assess the efficacy of EPA vs EPA/DHA compared to the standard preventive therapy across published RCTs investigating the use of n-3 PUFAs for primary or secondary prevention of CVD.

**Methods:**

Following a prespecified protocol registered in the PROSPERO database (CRD42023390587), we identified RCTs reporting CVD-attributable mortality in patients randomized to EPA, EPA/DHA, or a standard preventive therapy for primary or secondary CVD prevention. We used random effects meta-analysis to estimate pooled HRs of CVD-attributable mortality achieved with EPA or EPA/DHA relative to the standard preventive therapy.

**Results:**

Sixteen RCTs met the inclusion criteria, representing 127,771 patients in total (41% women, mean age 64 ± 5 years). Median follow-up was 3.7 years (IQR: 2.7-5.0 years). Compared to the standard preventive therapy, CVD-attributable mortality was significantly reduced with purified EPA (HR: 0.79 [95% CI: 0.67-0.94]; *P* = 0.006); this effect was less for EPA/DHA (HR: 0.92 [95% CI: 0.84-1.00]; *P* = 0.044).

**Conclusions:**

EPA lowered incident CVD-attributable mortality in RCTs investigating its use for primary or secondary CVD prevention. Relative to EPA, benefits reported with EPA/DHA were attenuated. Although more work is needed to understand these differences, EPA should preferentially be used in cardiovascular conditions for which it is indicated.

Purified eicosapentaenoic acid (EPA) and mixed eicosapentaenoic/docosahexaenoic acids (EPA/DHA), the major constituents of fish oil, are omega-3 (n-3) polyunsaturated fatty acids (PUFAs) of interest for their antiatherogenic effects in the prevention of cardiovascular disease (CVD).^1^ Several randomized clinical trials (RCTs) have studied the efficacy of EPA and EPA/DHA on reducing cardiovascular endpoints, including myocardial infarction (MI), stroke, and CVD-attributable mortality. Purified EPA in the form of icosapent ethyl has been endorsed as a Class IIa recommendation from the European Society of Cardiology, for the management of hypertriglyceridemia in high-risk patients.^2^ Landmark trials such as JELIS (Japan EPA Lipid Intervention Study)^3^ and REDUCE-IT (Reduction of Cardiovascular Events With Icosapent Ethyl-Intervention Trial)^4^ similarly led to a scientific advisory from the American Heart Association and approval by the U. S. Food and Drug Administration for the prescription-grade PUFA formulations in hypertriglyceridemia and also for cardiovascular risk reduction.^5^

Despite the well-established benefits of n-3 PUFAs in hypertriglyceridemia, confusion remains regarding their broader utility in primary or secondary prevention of CVD.^6^ The questions of efficacy and whether differences in study populations, drug formulations, and placebo arms may explain discrepant results of high-profile trials have been debated.^7^^,^^8^ Rigorous quantitative synthesis of the evolving literature from clinical endpoint trials is thus paramount to clarify evidence regarding the use of n-3 PUFAs in prevention and treatment of atherosclerosis. The number of trials continues to grow, most recently including results from RESPECT-EPA (Randomized Trial for Evaluation in Secondary Prevention Efficacy of Combination Therapy–Statin and Eicosapentaenoic Acid),^9^^,^^10^ which examined benefits of purified EPA for secondary prevention in Japanese patients with chronic coronary artery disease (CAD). In the current meta-analysis, we synthesize the latest available clinical trial data on purified EPA compared to mixed EPA/DHA as adjuncts to the standard preventive pharmacotherapy, focusing on the endpoint of CVD-attributable mortality.

## Materials and methods

### Literature search and article eligibility screening

We followed the Preferred Reporting Items for Systematic Reviews and Meta-Analyses guidelines throughout the design and conduct of our analysis. To minimize reporting bias, we prospectively registered a comprehensive literature search and analysis protocol to the PROSPERO database (CRD42023390587).^11^ We considered English-language published RCTs that randomized patients to receive purified EPA or mixed EPA/DHA vs placebo for the purposes of primary or secondary CVD prevention, and which reported CVD-attributable mortality (ie, adjudicated death from CVD) among control and treatment arms.

### Statistical analysis

Our primary endpoint of interest was the HR comparing rates of incident CVD-attributable mortality between patients receiving adjunctive EPA or EPA/DHA against control cohorts receiving the standard preventive therapies.^a^ For RCTs reporting CVD-attributable mortality but not reporting HRs, we estimated the trial-level HR as the ratio of CVD-attributable mortality rates between control and treatment groups during the trial follow-up period. We used random effects meta-analysis to obtain pooled HR estimates across all studies investigating purified EPA or mixed EPA/DHA. Pooled random effects estimates were obtained using restricted maximum likelihood estimators. Meta-analysis was performed in R using the *meta* and *metafor* packages.^13^^,^^14^

Full details of our literature search, article eligibility screening, data extraction, and statistical analysis are elaborated in the Supplemental Appendix.

### Ethical oversight

Our study received proper ethical oversight, but Institutional Review Board approval was not required for our study as no individual patient data were used to conduct the meta-analysis.

## Results

**Figure 1:**
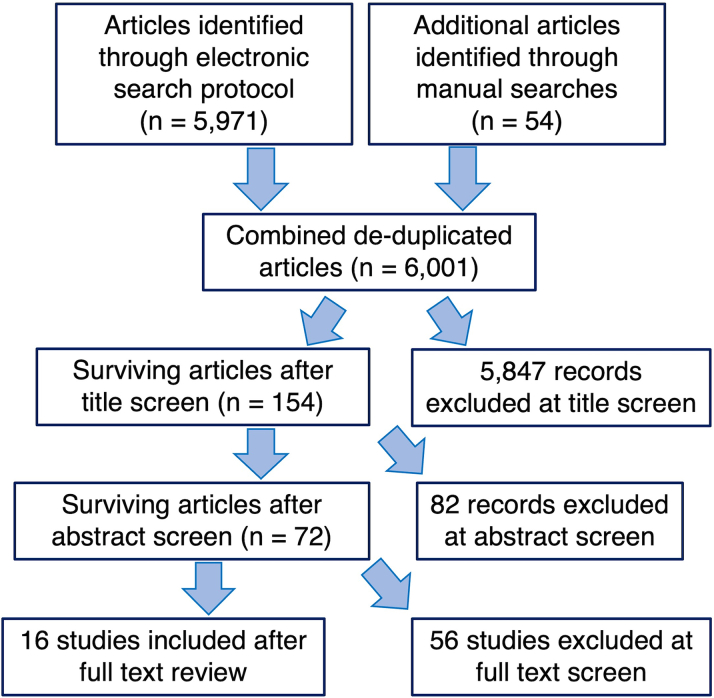
**Electronic Search and Article Eligibility Screen** This flow diagram summarizes our process of article identification and eligibility screening as detailed in our prospectively registered study protocol. We followed the Preferred Reporting Items for Systemic Reviews and Meta-Analyses guidelines in conducting study eligibility screening.

Our electronic search and manual bibliographic screen identified 5,971 potentially relevant publications; from which 16 RCTs were found to meet the eligibility criteria for the study inclusion (Figure 1).^3^^,^^4^^,^^10^^,^^12^^,^^15^, ^16^, ^17^, ^18^, ^19^, ^20^, ^21^, ^22^, ^23^, ^24^, ^25^, ^26^ Two RCTs randomized patients to adjunctive PUFA therapy for primary CVD prevention,^12^^,^^25^ 6 RCTs randomized patients for secondary prevention,^10^^,^^15^, ^16^, ^17^, ^18^, ^19^, ^20^, ^21^, ^22^ and 8 RCTs enrolled patients for either primary or secondary CVD prevention.^3^^,^^4^^,^^19^, ^20^, ^21^^,^^23^^,^^24^^,^^26^ Basic demographic and clinical data from the included RCTs are summarized in Table 1. Across all trials, 127,771 total patients were enrolled, 41% of whom were women. Across trials, the mean age at enrollment was 64 ± 5 years, and the median follow-up time was 3.7 years (IQR: 2.7-5.0 years).

**Table 1:** Randomized Clinical Trials of Mixed EPA/DHA or Purified EPA Versus Standard Preventive Therapy

Study/First Author	Year	Country	Patient Population	Primary or Secondary Prevention	N	Women	Age, y (mean ± SD)	Treatment^b^	Control	Follow-Up, y	Statistics Reported^a^
JELIS^3^	2007	Japan	HLD (TC ≥6.5 mmol/L) ± CAD	Both	18,645	12,786 (69%)	61 ± 9	EPA 1,800 mg, statin	Statin	4.6 ± 1.1	Mean ± SD
Nosaka et al^15^	2016	Japan	ACS undergoing emergent PCI	Secondary	238	56 (24%)	71 ± 11	EPA 1,800 mg, pitavastatin 2 mg	Pitavastatin 2 mg	1	-
REDUCE-IT^4^	2018	11 countries	Age ≥45 y; known CVD or DM + another CVD risk factor; on statin therapy; TG 135-499 mg/dL; LDL 41-100 mg/dL	Both	8,179	2,357 (29%)	63 ± 9	EPA 4,000 mg, statin ± ezetimibe	Mineral oil + statin ± ezetimibe	4.9	Median
RESPECT-EPA^10^	2024	Japan	Age 20-89 y; chronic CAD on statin therapy; EPA/AA < 0.4	Secondary	2,460	425 (17%)	67 ± 9	EPA 1,800 mg, statin	statin	5	Median
IEIS-4^16^	1997	India	Suspected acute MI; presentation ≤24 hours after symptom onset	Secondary	240	16 (7%)	49 ± 7	EPA 1,080 mg, DHA 720 mg	Aluminum hydroxide 100 mg	1	-
GISSI-P^17^	1999	Italy	Recent MI (≤3 mo); no CHF; no cancer; favorable short-term prognosis	Secondary	5,664	854 (15%)	59 ± 11	EPA 290 mg, DHA 580 mg	None (open-label)	3.5	-
Nilsen et al^18^	2001	Norway	Age ≥18 y, recent MI (4-8 d); less than NYHA functional class IV CHF symptoms; no cancer; no active GI bleed/ulcer; PLT>100,000/L; no liver insufficiency	Secondary	300	62 (21%)	64 ± 11	EPA 290 mg, DHA 580 mg	Corn oil 870 mg	1.5 (1 d - 2 y)	Median (range)
Raitt et al^19^	2005	USA	Patients planned for ICD placement for ECG-confirmed sustained VT/VF not due to acute MI or reversible cause, or with pre-existing ICD and recent (≤3 mo) ICD therapy for electrogram-confirmed VT/VF	Both	200	28 (14%)	63 ± 13	EPA 760 mg, DHA 540 mg	Olive oil 1,800 mg	2.0 (20 d - 2.3 y)	Median (range)
DO IT^20^	2010	Norway	Men aged 65-75 y; prior baseline total cholesterol 6.9–9.0 mmol/L & SBP <150 mm Hg; current SBP ≤170 mm Hg; current DBP ≤110 mm Hg; no cancer, ESRD, or chronic alcoholism	Both	563	0 (0%)	70 ± 3	EPA 1,180 mg, DHA 840 mg ± dietary counseling	Corn oil ± dietary counseling	3	-
GISSI-HF^21^	2008	Italy	Age >18; CHF from any cause with NYHA functional class II-IV symptom; Recently measured (≤3 mo) LVEF. If LVEF >40%, at least one CHF hospitalization in past year; no non-cardiac prognosis-limiting comorbidity (eg, cancer); no recent (≤1 mo) investigational therapy; no recent (≤1 mo) coronary revascularization procedure; no significant liver disease; if female: nonpregnant, nonlactating,with adequate birth control	Both	6,975	1,516 (22%)	67 ± 11	EPA 290 mg, DHA 580 mg, rosuvastatin 10 mg	Placebo capsule ± rosuvastatin 10 mg	3.9 (3.0-4.5)	Median (IQR)
Alpha Omega^22^	2010	Netherlands	Age 60-80 y; prior MI (≤10 y); daily margarine consumption ≥10 g; no baseline use of n-3 PUFA supplements at time of enrollment; no unintended weight loss >5 kg in past year; no cancer diagnosis with prognosis <1 year	Secondary	4,837	1,054 (22%)	69 ± 6	Margarine containing EPA 226 mg, DHA 150 mg ± ALA	Placebo margarine or ALA-containing margarine	3.4 (3.1-3.5)	Median (IQR)
ORIGIN^23^	2012	40 countries	Age ≥50 y; DM or impaired glucose tolerance; history of MI, stroke, or revascularization; angina with documented ischemia; urine albumin/creatinine >30 mg/g; left ventricular hypertrophy; 50% or more stenosis of a coronary, carotid, or lower-lib artery on angiography; or an ankle-brachial index <0.9; A1c <9%; no CABG within 4 y with no interval cardiovascular event; no severe heart failure; no prognosis-limiting cancer	Both	12,536	4,386 (35%)	64 ± 8	EPA 465 mg, DHA 375 mg	Olive oil 1,000 mg	6.2	Median
Risk and Prevention^24^	2013	Italy	Individuals with at least one of the following: Multiple CVD risk factors (4 of the following or DM plus one or more of the following: Age ≥65, male sex, HTN, HLD, current smoker, obesity (BMI ≥30), or family history of premature CVD), clinically evident CVD, or any condition placing patient at high CVD risk in opion of patient’s general practitioner; no prior MI; nonpregnant; no adverse short-term prognosis	Both	12,505	4,818 (39%)	64 ± 9	EPA 500 mg, DHA 420 mg	Olive oil 1,000 mg	5.0 (4.0-5.5)	Median (IQR)
ASCEND^12^	2018	UK	Age ≥40 y; DM; no evident CVD	Primary	15,480	5,796 (37%)	63 ± 9	EPA 460 mg, DHA 380 mg	Olive oil 1,000 mg	7.4	Mean
VITAL^25^	2018	USA	Men age ≥50 y or women ≥55 y; no prior CVD	Primary	25,871	13,085 (51%)	67 ± 7	EPA 460 mg, DHA 380 mg	Placebo capsule	5.3 (3.8-6.1)	Median (range)
STRENGTH^26^	2020	22 countries	Age ≥18 y at high CVD risk established CVD; DM and age ≥40 y if male or ≥50 y if female with at least 1 additional risk factor including chronic smoking, HTN, hsCRP ≥2 mg/L, or moderate-range albuminuria; or high-risk primary prevention patients aged ≥50 if male or ≥ 60 if female with at least 1 additional risk factor including family history of premature CAD, chronic smoking, hsCRP ≥2 mg/L, low GFR, or CAC score >300 AU.	Both	13,078	4,568 (35%)	63 ± 9	EPA 2200 mg, DHA 800 mg, statin	Corn oil, statin	3.5 (3.1-4.0)	Median (IQR)

AA = arachidonic acid; ACS = acute coronary syndrome; ALA, alpha-linoleic acid; ASCEND = A Study of Cardiovascular Events in Diabetes; AU = Agatston units; BMI = body mass index; CABG = coronary artery bypass graft; CAC = coronary artery calcium; CAD = coronary artery disease; CHF = congestive heart failure; CVD = cardiovascular disease; DBP = diastolic blood pressure; DHA = docosahexaenoic acid; DM = diabetes mellitus; DO IT = Diet and Omega-3 Intervention Trial; ECG = electrocardiogram; EPA = eicosapentaenoic acid; ESRD = end-stage renal disease; F/U = follow up; GDMT = goal-directed medical therapy; GFR = glomerular filtration rate; GI = gastrointestinal; GISSI-HF = effect of n-3 polyunsaturated fatty acids in patients with chronic heart failure; GISSI-P = dietary supplementation with n-3 polyunsaturated fatty acids and vitamin E after myocardial infarction; HLD = hyperlipidemia; hsCRP = high-sensitivity C-reactive protein; HTN = hypertension; ICD = implantable cardioverter-defibrillator; IEIS-4 = Indian Experiment of Infarct Survival–4; IPE = icosapent ethyl; JELIS = Japan EPA Lipid Intervention Study; LDL = low-density lipoprotein; LVEF = left ventricular ejection fraction; MI = myocardial infarction; ORIGIN = Outcome Reduction with an Initial Glargine Intervention; PCI = percutaneous coronary intervention; PLT = platelet; Pts = patients; PUFA = polyunsaturated fatty acid; REDUCE-IT = Reduction of Cardiovascular Events With Icosapent Ethyl-Intervention Trial; RESPECT-EPA = Randomized Trial for Evaluation in Secondary Prevention Efficacy of Combination Therapy–Statin and Eicosapentaenoic Acid; SBP = systolic blood pressure; TC = total cholesterol; TG = triglycerides; VF = ventricular fibrillation; VITAL = VITamin D and OmegA-3 TriaL; VT = ventricular tachycardia.

a Unless otherwise reported, studies had uniform follow-up time across all patients.

b EPA and DHA doses are approximate.

**Figure 2:**
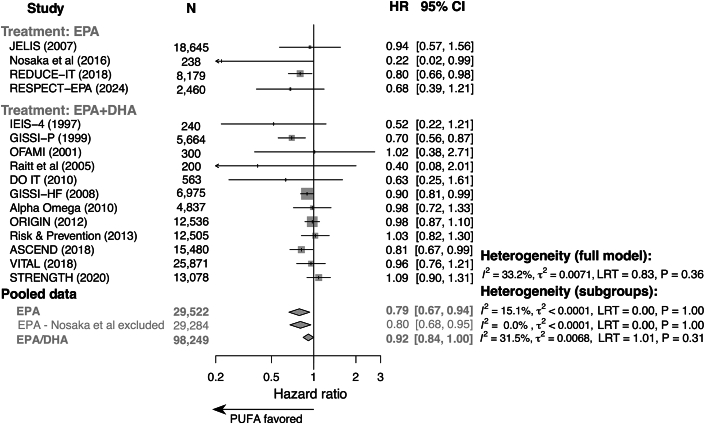
**Forest Plot Analysis of EPA and EPA/DHA Treatment Effects** Forest plots summarize trial-level and pooled HRs for CVD-attributable mortality for purified EPA (top), purified EPA with omission of the study by Nosaka et al. (middle), and mixed EPA/DHA (bottom) vs standard preventive therapy. ASCEND = A Study of Cardiovascular Events in Diabetes; CVD = cardiovascular disease; DHA = docosahexaenoic acid; DO IT = Diet and Omega-3 Intervention Trial; EPA = eicosapentaenoic acid; GISSI-HF = effect of n-3 polyunsaturated fatty acids in patients with chronic heart failure; GISSI-P = dietary supplementation with n-3 polyunsaturated fatty acids and vitamin E after myocardial infarction; IEIS-4 = Indian Experiment of Infarct Survival–4; JELIS = Japan EPA Lipid Intervention Study; ORIGIN = Outcome Reduction with an Initial Glargine Intervention; PUFA = polyunsaturated fatty acid; RCT = randomized clinical trial; REDUCE-IT = Reduction of Cardiovascular Events With Icosapent Ethyl–Intervention Trial; RESPECT-EPA = Randomized Trial for Evaluation in Secondary Prevention Efficacy of Combination Therapy–Statin and Eicosapentaenoic Acid; VITAL = VITamin D and OmegA-3 TriaL.

Pooled HRs estimated using traditional random effects meta-analysis supported efficacy of mixed EPA/DHA and purified EPA relative to standard pharmacotherapy in reducing CVD-attributable mortality (Figure 2). Compared to standard preventive therapy, marginally lower incident CVD-attributable mortality was observed in patients treated with mixed EPA/DHA (HR: 0.92 [95% CI: 0.84-1.00]; *P* = 0.044). Treatment with purified EPA was associated with robust reductions in CVD-attributable mortality relative to standard therapy (HR: 0.79 [95% CI: 0.67-0.94]; *P* = 0.006). Among studies of purified EPA, the RCT by Nosaka et al^15^ was identified as a potential outlier; this study reported an HR of only 0.22 with wide confidence bounds (95% CI: 0.02-0.99). However, the reduction in CVD-attributable mortality observed with purified EPA remained robust after omitting the RCT by Nosaka et al from the meta-analysis (HR: 0.80 [95% CI: 0.68-0.95]; *P* = 0.012). Our full random effects model found no difference in pooled treatment effects (ie, pooled HR) achieved with purified EPA vs mixed EPA/DHA as compared to the standard preventive therapy (Q(1) = 2.3; *P* = 0.128). Similarly, there was no difference in pooled treatment effects between purified EPA and mixed EPA/DHA after exclusion of the EPA study by Nosaka et al (Q(1) = 1.8, *P* = 0.179). Cross-study heterogeneity statistics for these analyses are elaborated in Supplemental Table 1.

**Figure 3:**
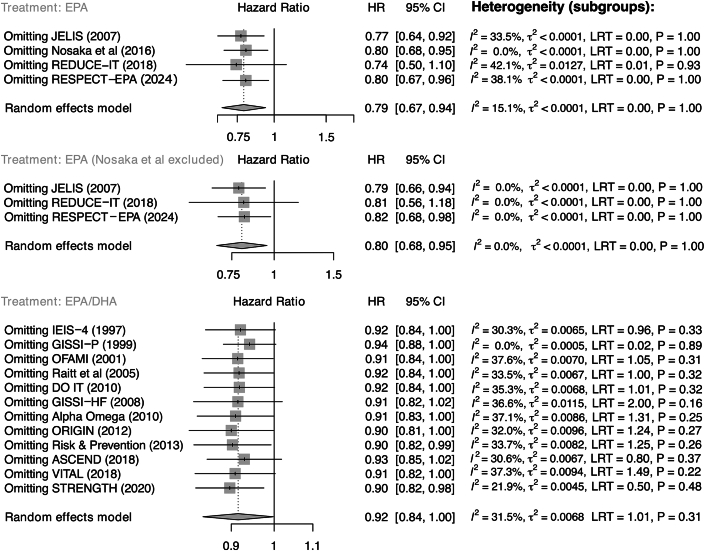
**Leave-One-Out Sensitivity Analysis** Forest plots summarize pooled HRs for CVD-attributable mortality for purified EPA (top), purified EPA with omission of the study by Nosaka et al (middle), and mixed EPA/DHA (bottom) vs standard preventive therapy, after omission of each individual RCT from analysis. Abbreviations as in Figure 2.

We next used leave-one-out sensitivity analyses to evaluate the influence of each individual RCT on pooled estimates (Figure 3). After omission of each individual RCT from analysis, we obtained comparable pooled HRs (purified EPA: HR: 0.74-0.80 vs 0.79 with all studies included; EPA/DHA: HR: 0.90-0.94 vs 0.92 with all studies included). Cross-study heterogeneity statistics for our sensitivity analyses are detailed in Supplemental Tables 2 to 4.

### Quality assessment/risk of bias analysis

Results of our quality assessment using the Cochrane Risk of Bias (RoB) 2 tool are summarized for each included study in Supplemental Table 5. Overall, on composite scoring across 5 domains, 8 studies (50%) were assessed to have low RoB, 7 studies (44%) had moderate concern for RoB, and 1 study (6%) scored high for RoB. The domains for which concerns were identified included “deviation from intended interventions” and “selection of reported results.” Meta-regressions of trial-level HRs against trial RoB scores are shown in Supplemental Figure 1. Meta-regression revealed a significant association between reported HRs and composite RoB scores for studies of EPA/DHA (β = −0.16 [−0.28 to −0.046]; *P* = 0.0064), but no significant association among studies of purified EPA (β = −0.15 [−0.56 to 0.42]; *P* = 0.77).

### Publication bias

**Figure 4:**
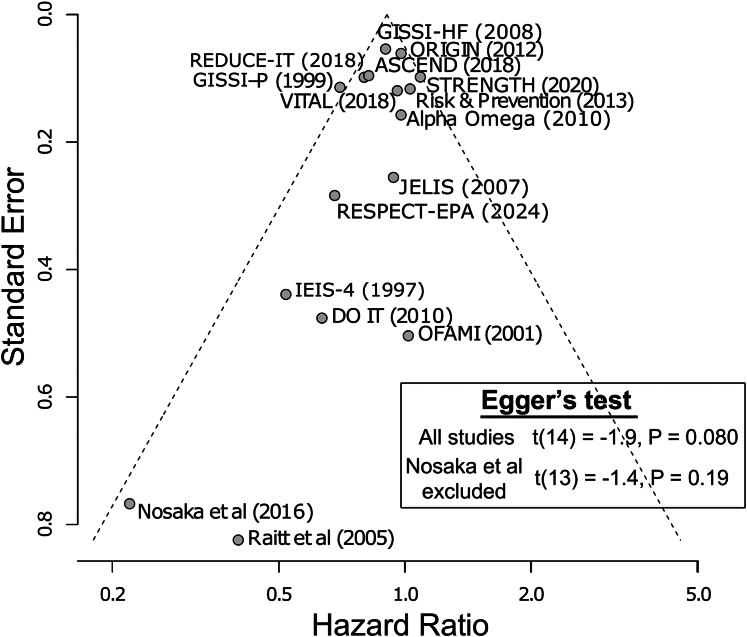
**Funnel Plot Analysis** Funnel plot summarizes the relationship between reported HRs of individual RCTs (horizontal axis) plotted against their respective trial-level standard errors (vertical axis). Slanted lines indicate 95% confidence bounds on HRs as a function of trial-level SE, assuming fixed treatment effects. Abbreviations as in Figure 2.

In addition, we performed funnel plot analysis to assess for systematic bias in the reporting of CVD-attributable mortality from individual RCTs, by regressing the reported HRs of each individual trial against their respective SEs (Figure 4). The reported trial-level HRs for 15 of 16 RCTs fell within 95% confidence bounds obtained under an assumption of fixed treatment effects. The only study falling outside these confidence bounds was dietary supplementation with n-3 polyunsaturated fatty acids and vitamin E after myocardial infarction, the first trial to study EPA/DHA. This trial began enrolling patients in 1993, before the availability of high-intensity statin therapy.^17^^,^^27^ The GISSI-P endpoint study yielded an HR of 0.70,^17^ which was below the 95% confidence bound for all studies under the assumption of fixed treatment effects. However, Egger test was not significant for funnel plot asymmetry whether including all studies (t[14] = −1.9; *P* = 0.080) or after omission of the study by Nosaka et al (t[13] = −1.4; *P* = 0.194).

### Supplementary analyses

All 4 purified EPA studies were conducted on patients receiving statin therapy. However, most of the EPA/DHA studies did not involve ubiquitous use of statins. Therefore, for the EPA/DHA studies, we reanalyzed data obtained exclusively in patients on statin therapy. This was limited to the STRENGTH trial and a subset of patients from the VITAL (VITamin D and OmegA-3 TriaL) trial (Supplemental Figure 2). The pooled HR for CVD-attributably mortality among these two EPA/DHA cohorts was not significant (HR: 0.99 [95% CI: 0.81-1.22]; *P* = 0.95). Heterogeneity statistics for this analysis are reported in Supplemental Table 6.

We further considered treatment effects on cardiovascular mortality in patients randomized to n-3 PUFA or the standard therapy specifically for either primary or secondary CVD prevention. Only 3 studies reported data within primary prevention cohorts, including JELIS for EPA and ASCEND (A Study of Cardiovascular Events in Diabetes) and VITAL for EPA/DHA. Three studies—JELIS, Nosaka et al and RESPECT-EPA—reported endpoint data on secondary prevention cohorts for purified EPA, and 5 studies reported endpoint data on secondary prevention cohorts for EPA/DHA. Forest plots summarizing results in primary and secondary prevention cohorts are shown in Supplemental Figures 3 and 4. Pooled HRs showed no significant effect on CVD-attributable mortality among primary prevention cohorts (EPA: HR: 1.10 [95% CI: 0.47-2.57]; *P* = 0.83; EPA/DHA: HR: 0.87 [95% CI: 0.75-1.02]; *P* = 0.078) or secondary prevention cohorts (EPA: HR: 0.69 [95% CI: 0.46-1.04]; *P* = 0.074; EPA/DHA: HR: 0.88 [95% CI: 0.68-1.14]; *P* = 0.33). Similarly, HRs for primary prevention cohorts receiving purified EPA were not significant after exclusion of the study by Nosaka et al (HR: 0.76 [95% CI: 0.50-1.15]; *P* = 0.19). Cross-study heterogeneity statistics among primary and secondary prevention cohorts are elaborated in Supplemental Tables 7 and 8.

## Discussion

Over the past 5 years, several meta-analyses have attempted to synthesize the published clinical endpoint literature to evaluate efficacy of n-3 PUFAs in preventing major adverse cardiovascular events or mortality (summarized by Bosomworth et al).^28^, ^29^, ^30^, ^31^, ^32^, ^33^, ^34^, ^35^ In 2019, Hu et al reported reductions in incident rates of MI, CVD events, and CVD-attributable mortality.^30^ In a 2020 meta-analysis of RCTs, Casula et al found reductions in MI, CVD events, and CVD-attributable mortality, and reported greater reductions with purified EPA.^29^ Doshi et al (2020) focused exclusively on RCTs of purified EPA, and found significant reductions in incident MI and hard CVD events.^36^ Also in 2020, Abdelhamid et al published an exhaustive Cochrane review on RCTs of n-3 PUFA supplementation, finding reductions in CAD events and CAD-attributable mortality.^28^ This Cochrane review found robust benefits for patients at either low or high CVD risk, as well as a dose-dependent response for CAD events and CAD-attributable mortality.^28^^,^^35^ In 2021, Bernasconi et al reported 13% reduction in incident MI, 35% reduction in fatal MI, and 9% reduction in CAD-attributable mortality across RCTs of EPA and EPA/DHA, with benefits dependent on the dose of n-3 PUFAs administered.^32^ These results were mirrored in another 2021 meta-analysis by Khan et al, which incorporated 2 major RCTs (STRENGTH^26^ and OMEMI [Omega-3 Fatty acids in Elderly with Myocardial Infarction]^37^) reporting negative endpoint results with mixed EPA/DHA formulations.^34^ More recently, this pattern of results was once again recapitulated in meta-analyses by Yu et al^33^ and Shen et al,^31^ who likewise found that n-3 PUFA supplementation achieved reductions in hard CVD events and CVD-attributable mortality.

Notably, we found more robust benefits for cardiovascular mortality with purified EPA relative to mixed EPA/DHA formulations. The mechanisms for the greater reduction in CVD-attributable mortality achieved with EPA compared to EPA/DHA in our study are not fully understood. However, a recent retrospective study of 987 randomly selected patients from the INSPIRE biobank registry suggested that higher DHA serum levels may blunt beneficial actions of EPA, and are associated with higher incidence of adverse cardiovascular events when serum EPA levels are low.^38^ Moreover, a broad literature examining effects of EPA vs EPA/DHA at the molecular and cellular levels,^39^, ^40^, ^41^, ^42^ their effects at the level of atherosclerotic plaque quantifiable with clinical imaging,^8^ and their effects at the clinical endpoint level evidenced in the RCT literature reviewed here, all suggest more robust benefit with purified EPA rather than mixed EPA/DHA.

A criticism raised by Nissen et al pertaining to benefits of purified EPA is the use of mineral oil as a placebo comparator in the REDUCE-IT trial.^26^ This critique notes rises in C-reactive protein and other biomarkers associated with atherosclerosis among the REDUCE-IT patients who received mineral oil, relative to patients randomized to EPA.^4^^,^^7^ However, the theoretic possibility of confounding by mineral oil comparator in REDUCE-IT does not explain findings suggesting dose-dependent benefit of EPA in the broader literature.^43^ Moreover, we obtained comparable results with a pooled HR of 0.74 (95% CI: 0.50-1.10) for cardiovascular mortality reduction among purified EPA studies after excluding REDUCE-IT from analysis entirely, compared to an HR of 0.79 (95% CI: 0.67-0.94) with REDUCE-IT included. Overall, multiple national regulatory agencies (including, among others, the U.S. Food and Drug Administration, European Medicines Agency, and Health Canada) have examined the evidence surrounding this debate and have each come to their own conclusion that meaningful benefits exist for the population at risk, and have approved icosapent ethyl (ie, EPA) for their respective countries.

### Study limitations

An important limitation of our work concerns the fact that CVD-attributable mortality was reported as a secondary rather than primary endpoint in all but one reviewed trial (ORIGIN [Outcome Reduction with an Initial Glargine Intervention]). We also included data from studies reporting CVD-attributable mortality even in cases where the primary endpoint was negative. This raises a theoretic RoB, particularly if CVD-attributable mortality was not a prespecified endpoint in each trial. However, given the lack of studies reporting CVD-attributable mortality as a primary endpoint, the only tractable approach was to consider all trials reporting CVD-attributable mortality regardless of whether reported as a primary or secondary endpoint, and regardless of whether the primary endpoint was significant.

There were further differences in the design of the EPA trials that may influence results. REDUCE-IT was the only double-blinded and international trial on EPA. In contrast, JELIS, RESPECT-EPA, and the trial by Nosaka et al were open-label Japanese trials with no placebo comparator and with patients unblinded to therapy. The use of open-label designs in these trials presents a RoB and could have influenced our results. Furthermore, there were important differences in EPA dose administered across trials. Japanese studies used 1.8 g/day of EPA, compared to 4 g/day in REDUCE-IT. However, Japanese patients also have higher dietary consumption of n-3 PUFAs compared to Western patients. When assessing for a potential correlation between the administered EPA dose and treatment effect across studies of purified EPA, we found no significant association (β = 0.0000 [−0.0002 to 0.0003]; *P* = 0.77). It may be that comparable benefits are achieved in Japanese patients at lower doses of EPA given their increased dietary consumption of n-3 PUFAs relative to Western patients, but this requires further investigation.

Several other factors also limit the interpretation of our results. Our primary analysis combined patients receiving n-3 PUFA therapy for primary and secondary CVD prevention. We performed a subgroup analysis restricted to patients on therapy for either primary or secondary prevention, but the pooled HRs in this analysis were not significant. Lack of significance within these subgroups may relate to a lack of statistical power, and more research is needed within primary and secondary prevention groups specifically to assess which patients derive most benefit. In addition to differences in clinical presentation, there were also important differences in follow-up, concomitant statin therapy, use of other adjunctive pharmacotherapies, duration of therapy, and baseline risk profiles. We also performed a range of statistical quality analyses in this meta-analysis, which we further discuss in the Supplemental Appendix.

Finally, it is important to note that contemporary high-intensity statin formulations achieve greater low-density lipoprotein–lowering effects and cardiovascular endpoint reduction than were used during the first mixed EPA/DHA trials, such as GISSI-P.^17^^,^^27^^,^^44^ In fact, only 2 of the 12 reviewed EPA/DHA trials reported our endpoint of interest for cohorts exclusively receiving statin therapy, including STRENGTH and a subset of patients from VITAL. Among these cohorts with ubiquitous statin use, EPA/DHA showed no benefit. The fact that RCTs of mixed EPA/DHA formulations achieved underwhelming efficacy compared to purified EPA despite their lack of consistent statin use speaks even more strongly to the greater success observed with purified EPA in reducing adverse cardiovascular events and attributable mortality. Despite an extensive literature investigating EPA/DHA for cardiovascular risk reduction, unlike purified EPA no RCT has ever demonstrated significant reduction in cardiovascular events with EPA/DHA as adjunct to a high-intensity statin therapy.^1^

## Conclusions

In this most up-to-date analysis synthesizing data from 127,771 patients, we focused on the endpoint of CVD-attributable mortality, incorporating new data from 2,460 patients in the RESPECT-EPA trial.^10^ Using a prespecified and published protocol for study search and eligibility screening,^11^ we identified 16 RCTs reporting hard endpoint data on CVD-attributable mortality achieved with n-3 PUFA as compared to standard preventive therapy. Four RCTs meeting our inclusion criteria compared CVD-attributable mortality between purified EPA and standard therapy alone, and 12 RCTs reported CVD-attributable mortality with mixed EPA/DHA compared to the standard therapy. In our pooled analysis, patients receiving both mixed EPA/DHA and purified EPA had significantly lower CVD-attributable mortality compared to standard preventive therapy alone. However, despite markedly more statistical power encompassing a cohort of 98,249 patients spanning 12 RCTs, mixed EPA/DHA achieved only modest reductions in CVD-attributable mortality (pooled HR: 0.92; *P* = 0.044). Critically, EPA/DHA results were correlated with study RoB, and there was no evidence of reduction in CVD-attributable mortality with EPA/DHA among cohorts on statin therapy. In contrast, the efficacy of purified EPA was investigated in 4 RCTs meeting the inclusion criteria. This smaller cohort encompassed 29,522 Japanese or international patients, all on statin therapy, and collectively demonstrated robust reduction in CVD-attributable mortality (pooled HR: 0.79; *P* = 0.006). Consistent with a wealth of insights spanning the molecular and cellular levels as well as the coronary imaging literature, our results demonstrate that purified EPA offers more robust benefits than mixed EPA/DHA for reducing CVD-attributable mortality (Central Illustration).

Perspectives **COMPETENCY IN MEDICAL KNOWLEDGE:** Among 12 RCTs, mixed EPA/DHA modestly reduced cardiovascular mortality (HR: 0.92 [95% CI: 0.84-1.00]; *P* = 0.044). However, among 2 RCTs reporting data restricted to patients on statin therapy, EPA/DHA achieved no reduction in cardiovascular mortality (HR: 0.99 [95% CI: 0.81-1.22]; *P* = 0.95). Among 4 RCTs, purified EPA robustly reduced cardiovascular mortality compared to statin therapy alone (HR: 0.79 [95% CI: 0.67-0.94]; *P* = 0.006).

**COMPETENCY IN PATIENT CARE:** EPA should be used preferentially to mixed EPA/DHA in cardiovascular conditions for which it is indicated.

**TRANSLATIONAL OUTLOOK:** More research is needed at the basic and translational science levels to clarify the mechanistic basis for differences between EPA and EPA/DHA in cardiovascular risk reduction.

**Central Illustration:**
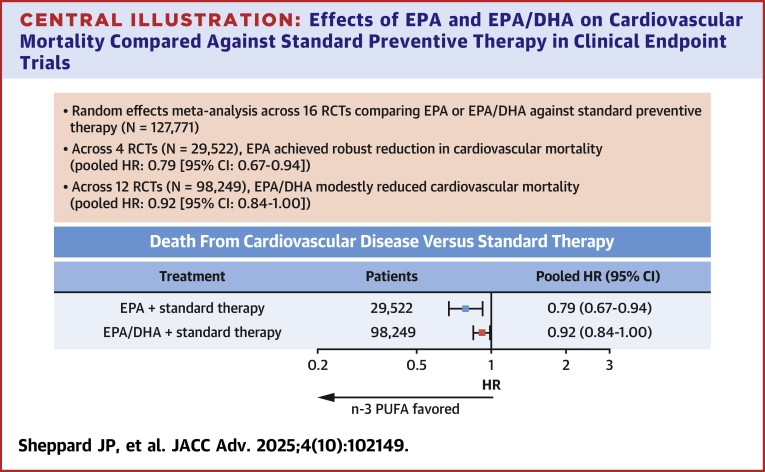
**Effects of EPA and EPA/DHA on Cardiovascular Mortality Compared Against Standard Preventive Therapy in Clinical Endpoint Trials** Our random effects meta-analysis of published randomized controlled trials demonstrated robust reduction in cardiovascular mortality with purified EPA but only modest reduction in cardiovascular mortality with mixed EPA/DHA when compared to standard preventive therapy. EPA = eicosapentaenoic acid; DHA = docosahexaenoic acid; RCT = randomized clinical trial.

## Funding support and author disclosures

Dr Bhatt is on the advisory board of Angiowave, Bayer, Boehringer Ingelheim, CellProthera, Cereno Scientific, E-Star Biotech, High Enroll, Janssen, Level Ex, McKinsey, Medscape Cardiology, Merck, NirvaMed, Novo Nordisk, Stasys, and Tourmaline Bio; Board of Directors: American Heart Association New York City, Angiowave (stock options), Bristol Myers Squibb (stock), DRS.LINQ (stock options), and High Enroll (stock); Consultant: Broadview Ventures, Corcept Therapeutics, GlaxoSmithKline, Hims, SFJ, Summa Therapeutics, and Youngene; Data Monitoring Committees: Acesion Pharma, Assistance Publique-Hôpitaux de Paris, Baim Institute for Clinical Research (formerly Harvard Clinical Research Institute, for the PORTICO trial, funded by 10.13039/100006279St. Jude Medical, now Abbott), 10.13039/100008497Boston Scientific (Chair, PEITHO trial), 10.13039/100012380Cleveland Clinic, Contego Medical (Chair, PERFORMANCE 2), 10.13039/100006513Duke Clinical Research Institute, 10.13039/100000871Mayo Clinic, Mount Sinai School of Medicine (for the ENVISAGE trial, funded by 10.13039/501100022274Daiichi Sankyo, Japan; for the ABILITY-DM trial, funded by Concept Medical; for ALLAY-HF, funded by Alleviant Medical), Novartis, Population Health Research Institute, and Rutgers University (for the NIH-funded MINT Trial); Honoraria: American College of Cardiology (Senior Associate Editor, Clinical Trials and News, ACC.org; Chair, ACC Accreditation Oversight Committee), Arnold and Porter law firm (work related to Sanofi/Bristol-Myers Squibb clopidogrel litigation), Baim Institute for Clinical Research (formerly Harvard Clinical Research Institute; AEGIS-II executive committee funded by 10.13039/100008322CSL Behring, Australia), Belvoir Publications (Editor in Chief, Harvard Heart Letter), Canadian Medical and Surgical Knowledge Translation Research Group (clinical trial steering committees), CSL Behring (AHA lecture), Cowen and Company, Duke Clinical Research Institute (clinical trial steering committees, including for the PRONOUNCE trial, funded by 10.13039/501100003122Ferring Pharmaceuticals), HMP Global (Editor in Chief, Journal of Invasive Cardiology), Journal of the American College of Cardiology (Guest Editor; Associate Editor), Level Ex, Medtelligence/ReachMD (CME steering committees), MJH Life Sciences, Oakstone CME (Course Director, Comprehensive Review of Interventional Cardiology), Piper Sandler, Population Health Research Institute (for the COMPASS operations committee, publications committee, steering committee, and USA national co-leader, funded by 10.13039/100004326Bayer), WebMD (CME steering committees), and Wiley (steering committee); Other: Clinical Cardiology (Deputy Editor); Patent: Sotagliflozin (named on a patent for sotagliflozin assigned to Brigham and Women's Hospital who assigned to Lexicon; neither I nor Brigham and Women's Hospital receive any income from this patent); Research Funding: Abbott, Acesion Pharma, Afimmune, Aker Biomarine, Alnylam, Amarin, Amgen, AstraZeneca, Bayer, Beren, Boehringer Ingelheim, Boston Scientific, Bristol-Myers Squibb, Cardax, CellProthera, Cereno Scientific, Chiesi, CinCor, Cleerly, CSL Behring, Faraday Pharmaceuticals, Ferring Pharmaceuticals, Fractyl, Garmin, HLS Therapeutics, Idorsia, Ironwood, Ischemix, Janssen, Javelin, Lexicon, Lilly, Medtronic, Merck, Moderna, MyoKardia, NirvaMed, Novartis, Novo Nordisk, Otsuka, Owkin, Pfizer, PhaseBio, PLx Pharma, Recardio, Regeneron, Reid Hoffman Foundation, Roche, Sanofi, Stasys, Synaptic, The Medicines Company, Youngene, and 89Bio; Royalties: Elsevier (Editor, Braunwald’s Heart Disease); Site Co-Investigator: Cleerly. Dr Budoff is a consultant for General Electric. Dr Nelson serves on the Speakers’ Bureaus for Amarin Corporation, Esperion Therapeutics, Regeneron Pharmaceuticals, and Amgen; he also holds stock in Amgen. All other authors have reported that they have no relationships relevant to the contents of this paper to disclose.

## Footnotes

a In the case of the ASCEND trial, we used their reported endpoint of vascular death, which included death from myocardial infarction, sudden cardiac death, pulmonary embolism, or ischemic stroke, but excluded death from intracranial hemorrhage.^12^
