# Supplementary material for: Effects of Eicosapentaenoic Acid vs Eicosapentaenoic/Docosahexaenoic Acids on Cardiovascular Mortality: Meta-Analysis of Clinical Trials
Source: JACC Adv. 2025 Sep 19;4(10):102149. doi: 10.1016/j.jacadv.2025.102149 (PMC12791848; doi:10.1016/j.jacadv.2025.102149)
Supplement: Supplemental_Material [file mmc1.docx]

**SUPPLEMENTAL APPENDIX**

**SUPPLEMENTAL METHODS**

*Literature Search and Article Eligibility Screening*

Our automated electronic search was limited to the PubMed database maintained by the National Library of Medicine (<https://pubmed.ncbi.nlm.nih.gov>). The PubMed search consisted of the following intersectional search for key text:

*(“omega 3 fatty acid” OR “omega 3 fatty acids” OR “omega-3 fatty acids” OR “polyunsaturated fatty acid” OR “PUFA” OR “eicosapentaenoic acid” OR “docosahexaenoic acid” OR “EPA/DHA”) AND (“cardiovascular endpoint” OR “cardiovascular disease” OR “cardiovascular mortality” OR “cerebrovascular accident” OR “stroke” OR “myocardial infarction” OR “coronary heart disease”)*

This text search strategy was based upon a PubMed search strategy described in a prior meta-analysis on this topic by Khan *et al.*, with modifications.^1^ The final electronic search used for article eligibility screening was performed on April 27, 2023. Three authors (LP, TD, and JB) independently screened article titles for potential inclusion, with any disagreements reviewed and adjudicated by author JPS. The next stage consisted of screening abstracts of articles surviving the title screen and was conducted independently by authors LP and JPS to ensure consensus. Cases of discordance were reviewed and adjudicated by JPS. Finally, full texts of articles surviving the abstract screen were reviewed independently by LP and JPS for eligibility. Cases of discordance were reviewed and adjudicated by JPS, who made the final determination on article inclusion. Separately, manual bibliographic searches were performed during the early stages of research, which included the use of the Google search engine and Google Scholar. The only research study meeting our eligibility criteria not initially captured in our automated PubMed search strategy was the RESPECT-EPA trial, which was as yet unpublished in journal article form at the time of search. This trial had been presented at a national meeting by the time of our search, and was subsequently published in journal article form online on June 14, 2024 and in print on August 6, 2024.^2^

*Data Extraction*

We extracted data on patient demographics, clinical presentation, details of statin and n-3 PUFA therapies, placebo comparators, and follow-up. Specific data elements extracted included the year of study publication; country of study origin (or if international, the number of countries represented); a brief narrative description of the patient population (e.g, Men > 50 and Women > 55 without prior CVD); whether the study investigated n-3 PUFA usage in primary CVD prevention (patients without known CVD), secondary prevention (patients with prior CVD), or both; total number of patients enrolled; number of women enrolled; patient age at enrollment; details of n-3 PUFA treatment including daily dosage of EPA and/or DHA; details of statin therapy; details of any placebo comparator; and clinical follow-up time. These data elements were tabulated in an Excel spreadsheet to facilitate statistical analysis. We did not contact authors of any of the reviewed studies for data not reported in their original publications.

***Statistical Analysis***

Our pre-specified analysis consisted of a comparison of the treatment effects of purified EPA versus mixed EPA/DHA on cardiovascular mortality (death attributable to cardiovascular disease), as compared to standard preventive therapies.^3^ Treatment effects were assessed in terms of study-level hazard ratios; when not directly reported, rate ratios of cardiovascular mortality between n-3 PUFA and control arms were accepted as a surrogate measure in lieu of the hazard ratio. We also conducted pre-specified subgroup analyses of published endpoint data, stratified by whether the enrolled patients were receiving therapy for primary or secondary CVD prevention.

We also reported *post-hoc*, exploratory analyses. First, we re-analyzed pooled outcomes within EPA/DHA cohorts universally on statin therapy. Second, we re-analyzed pooled outcomes after exclusion of the purified EPA trial by Nosaka et al.^4^ We were concerned by this trial’s extreme reported hazard ratio of 0.22 for cardiovascular mortality, especially given its small sample size of 238 patients compared to other included trials.^4^ Thus, we sought to evaluate pooled effects with purified EPA after excluding this study entirely. However, there was no pre-specified statistical rationale for excluding the study by Nosaka et al., so this subgroup analysis was purely exploratory.

*Leave-One-Out Sensitivity Analysis*

To assess the influence of each individual study on our results, we performed a sensitivity analysis in which we recomputed pooled treatment effects with each individual RCT successively excluded from analysis. We performed this analysis both with and without the study by Nosaka et al. This analysis was performed to assess whether the results we obtained were contingent on results from individual trials.

*Quality Assessment / Risk of Bias Analysis*

To assess study quality and risk of bias, we used the Cochran Risk-of-Bias Version 2 tool (RoB 2), a structured questionnaire that spans five domains (randomization, deviation from intended interventions, missing outcome data, measurement of the outcome, and selection of the reported result) relevant to risk of study bias.^5^ This validated RoB metric was pre-specified in our prospectively registered study protocol.^3^ The RoB 2 questionnaire was completed by author JPS for all reviewed studies. We reported the domain subscores for each study, as well as the overall risk of bias “judgment” that serves as a composite risk of bias score across domains. Based on the domain scores, each study was assigned a composite RoB score as follows: (1) low, (2) some concerns, or (3) high risk of bias.^5^ To gauge whether risk of bias could influence our endpoint of interest, we performed meta-regression of the trial-level hazard ratios for cardiovascular mortality against each trial’s composite RoB 2 score as a regressor. We performed meta-regression separately among studies assessing EPA or EPA/DHA versus standard preventive therapy. Meta-regression was performed using the *meta* package for R.^6,7^

*Assessment of Publication Bias*

The presence of publication (or reporting) bias was assessed in our meta-analysis using funnel plot analysis, which assesses for systematic relationships between the treatment effects reported by individual trials (i.e., trial-level hazard ratios) and their respective standard errors. This analysis seeks to ensure that the pooled treatment effects for EPA or EPA/DHA were not skewed by relatively underpowered studies that selectively published more favorable treatment effects than reflected in the true underlying body of evidence. We quantified publication bias using the Egger’s regression test coefficient, which regresses the trial-level hazard ratios against their respective standard errors.^8^

*Assessment of Study Heterogeneity*

Cross-study heterogeneity in reported hazard ratios was quantified using the Cochran’s Q and *I^2^* coefficients as primary statistics. Cochran’s Q test uses a chi-square-based approach to test the hypothesis that the true treatment effect is the same across studies; Q is defined as the weighted sum of squared differences between individual trial effects and the pooled treatment effect, with statistical significance assessed based on a Chi-squared distribution, and P < 0.05 conveying significant heterogeneity.^9^ Cochran’s Q may underestimate heterogeneity when the number of studies is small, and overestimate heterogeneity when the number of studies is large. The I^2^ or inconsistency index is defined as *100% x (Q – df)/Q,* and describes the percentage of total variance attributable to cross-study heterogeneity as opposed to chance.^9,10^ Unlike Cochran’s Q, I^2^ does not depend on the number of included studies. Traditionally, I^2^ less than 25% is considered low heterogeneity, I^2^ between 25% and 50% is considered moderate, and I^2^ over 50% is considered high heterogeneity.^10^ As secondary heterogeneity statistics, we also report tau and tau^2^ coefficients, likelihood ratios, and likelihood ratio test (LRT) statistics. Tau^2^ estimates the between study variance, while tau has units equivalent to effect size. Likelihood ratios compare the likelihood of a random effects model to that of a fixed effects model. The LRT statistic is defined as LRT = -2 * ln(Likelihood of fixed effects model / Likelihood of random effects model), with significance assessed based on a Chi-squared distribution with one degree of freedom.

**SUPPLEMENTAL DISCUSSION**

*Sensitivity Analysis*

Our leave-one-out sensitivity analysis assessed CVD-attributable mortality after exclusion of each individual RCT from analysis. The pooled hazard ratio for CVD-attributable mortality with purified EPA was similar, but no longer reached significance after omission of the REDUCE-IT trial from analysis (HR = 0.74 [0.50, 1.10], P = 0.14). On the other hand, a significant reduction in CVD-attributable mortality with EPA/DHA was contingent on 7 of the 12 reviewed trials (58%), Pooled hazard ratios of CVD-attributable mortality with EPA/DHA no longer achieved significance after omission of IEIS-4 (HR = 0.92 [0.84, 1.00], P = 0.0587), GISSI-P (HR = 0.94 [0.88, 1.00], P = 0.0673), Raitt et al. (HR = 0.92 [0.84, 1.00], P = 0.0503), DO IT (HR = 0.92 [0.84, 1.00], P = 0.0534), GISSI-HF (HR = 0.91 [0.82, 1.02], P = 0.1098), ASCEND (HR = 0.93 [0.85, 1.02], P = 0.1177, and VITAL (HR = 0.91 [0.82, 1.00], P = 0.0569). The fact that significance of the pooled hazard ratio for EPA/DHA was so sensitive to individual study exclusions calls the robustness of the effect into question.

*Study Heterogeneity Analysis*

We quantified cross-study heterogeneity of the treatment effects for CVD-attributable mortality observed across trials. For our main analysis and sensitivity analyses, Cochran’s Q statistics were insignificant in all cases, but we further considered I^2^ values, considering the relatively low number of reviewed studies. For the full meta-analysis, overall heterogeneity fell into the moderate range with I^2^ of 33%. Heterogeneity among EPA trials was low, with I^2^ of 15%. Across mixed EPA/DHA trials, heterogeneity was moderate with I^2^ of 32%. Heterogeneity in our leave-one-out sensitivity analyses fell into the low to moderate range, with I^2^ values of 0-42% and 0-37% after individual study exclusions for EPA and EPA/DHA, respectively. Most cross-study heterogeneity was contributed by two outlying studies – Nosaka et al. for EPA and GISSI-P for EPA/DHA. After exclusion of these two studies, I^2^ of 0% was achieved among the remaining EPA and EPA/DHA subgroups. Overall, our choice of a random effects estimator was appropriate given the presence of non-negligible heterogeneity across all reviewed studies.

*Funnel Plot Analysis & Risk of Bias Analysis*

We investigated potential sources of bias both through funnel plot analysis and a formal risk of bias analysis. Egger’s tests assessing for funnel plot asymmetry failed to detect selective reporting of favorable treatment effects among the reviewed studies. However, our risk of bias analysis urges caution in interpreting the results, particularly with respect to the EPA/DHA studies. Here, we used a validated risk of bias metric (the Cochrane RoB 2 tool) and found that higher RoB score correlated with more favorable treatment effects among studies of EPA/DHA. Although we found no correlation between RoB score and hazard ratios among studies of EPA, there were only four EPA studies. Thus, we cannot fully exclude the possibility that study bias influenced results for EPA as well.

**SUPPLEMENTAL FIGURES**

**Supplemental Figure 1. Risk of bias analysis.** Bubble plots highlight relationships between study risk of bias score (horizontal axis) and trial-level hazard ratios (vertical axis) among studies of EPA (top) and EPA/DHA (bottom). Regression lines were fit using meta-regression.

**Supplemental Figure 2. Forest plot analysis of EPA/DHA treatment effects among patients on statin therapy.** Forest plot summarizes trial-level and pooled hazard ratios for CVD-attributable mortality for mixed EPA/DHA cohorts limited to patients exclusively receiving statin therapy.

**Supplemental Figure 3. Forest plot analysis of EPA and EPA/DHA treatment effects within primary CVD prevention cohorts**. Forest plots summarize trial-level and pooled hazard ratios for CVD-attributable mortality for purified EPA (top) and mixed EPA/DHA (bottom) versus standard preventive therapy, with analysis restricted to primary CVD prevention cohorts (i.e., patients without baseline CVD).

**Supplemental Figure 4. Forest plot analysis of EPA and EPA/DHA treatment effects within secondary CVD prevention cohorts**. Forest plots summarize trial-level and pooled hazard ratios for CVD-attributable mortality for purified EPA (top) and mixed EPA/DHA (bottom) versus standard preventive therapy, with analysis restricted to secondary CVD prevention cohorts (i.e., patients with baseline CVD).

**SUPPLEMENTAL TABLES**

**Supplemental Table 1.** Heterogeneity statistics for primary meta-analysis of reported cardiovascular mortality hazard ratios comparing EPA or EPA/DHA to standard preventive therapy.

| Pooled Analysis | DF | Q, P-value | I^2^ | Tau^2^ | Tau | Likelihood ratio test statistic, P-value | Likelihood ratio |
| --- | --- | --- | --- | --- | --- | --- | --- |
| Full Model | 15 | 22.44, P = 0.10 | 33.2% | 0.0071 | 0.0843 | 0.83, P = 0.36 | 1.51 |
| EPA | 3 | 3.54, P = 0.32 | 15.1% | <0.0001 | 0.0008 | 0.00, P = 1.00 | 1.00 |
| EPA/DHA | 11 | 16.06, P = 0.14 | 31.5% | 0.0068 | 0.0825 | 1.01, P = 0.31 | 1.66 |
| EPA (Nosaka excluded) | 2 | 0.73, P = 0.70 | 0.0% | <0.0001 | <0.0001 | 0.00, P = 1.00 | 1.00 |

**Supplemental Table 2.** Heterogeneity statistics for leave-one-out sensitivity analysis of cardiovascular mortality hazard ratios reported among studies comparing EPA to standard preventive therapy.

| EPA sensitivity analysis | DF | Q, P-value | I^2^ | Tau^2^ | Tau | Likelihood ratio test statistic, P-value | Likelihood ratio |
| --- | --- | --- | --- | --- | --- | --- | --- |
| JELIS excluded | 2 | 3.01, P = 0.22 | 33.5% | <0.0001 | 0.0022 | 0.00, P = 1.00 | 1.00 |
| Nosaka et al. excluded | 2 | 0.73, P = 0.70 | 0.0% | <0.0001 | <0.0001 | 0.00, P = 1.00 | 1.00 |
| REDUCE-IT excluded | 2 | 3.46, P = 0.18 | 42.1% | 0.0127 | 0.1128 | 0.01, P = 0.93 | 1.00 |
| RESPECT-EPA excluded | 2 | 3.23, P = 0.20 | 38.1% | <0.0001 | 0.0007 | 0.00, P = 1.00 | 1.00 |

**Supplemental Table 3.** Heterogeneity statistics for leave-one-out sensitivity analysis of cardiovascular mortality hazard ratios reported among studies comparing EPA to standard preventive therapy, with the study by Nosaka et al. excluded.

| EPA sensitivity analysis (Nosaka et al. excluded) | DF | Q, P-value | I^2^ | Tau^2^ | Tau | Likelihood ratio test statistic, P-value | Likelihood ratio |
| --- | --- | --- | --- | --- | --- | --- | --- |
| JELIS & Nosaka et al. excluded | 1 | 0.29, P = 0.59 | 0.0% | <0.0001 | <0.0001 | 0.00, P = 1.00 | 1.00 |
| REDUCE-IT & Nosaka et al. excluded | 1 | 0.72, P = 0.40 | 0.0% | <0.0001 | <0.0001 | 0.00, P = 1.00 | 1.00 |
| RESPECT-EPA & Nosaka et al. excluded | 1 | 0.35, P = 0.56 | 0.0% | <0.0001 | <0.0001 | 0.00, P = 1.00 | 1.00 |

**Supplemental Table 4.** Heterogeneity statistics for leave-one-out sensitivity analysis of cardiovascular mortality hazard ratios reported among studies comparing EPA/DHA to standard preventive therapy.

| EPA/DHA sensitivity analysis | DF | Q, P-value | I^2^ | Tau^2^ | Tau | Likelihood ratio test statistic, P-value | Likelihood ratio |
| --- | --- | --- | --- | --- | --- | --- | --- |
| IEIS-4 excluded | 10 | 14.36, P = 0.16 | 30.3% | 0.0065 | 0.0808 | 0.96, P = 0.33 | 1.62 |
| GISSI-P excluded | 10 | 9.79, P = 0.46 | 0.0% | 0.0005 | 0.0232 | 0.02, P = 0.89 | 1.01 |
| OFAMI excluded | 10 | 16.02, P = 0.099 | 37.6% | 0.0070 | 0.0839 | 1.05, P = 0.31 | 1.69 |
| Raitt et al excluded | 10 | 15.04, P = 0.13 | 33.5% | 0.0067 | 0.0820 | 1.00, P = 0.32 | 1.65 |
| DO IT excluded | 10 | 15.45, P = 0.12 | 35.3% | 0.0068 | 0.0825 | 1.01, P = 0.32 | 1.66 |
| GISSI-HF excluded | 10 | 15.78, P = 0.11 | 36.6% | 0.0115 | 0.1074 | 2.00, P = 0.16 | 2.71 |
| AlphaOmega excluded | 10 | 15.91, P = 0.10 | 37.1% | 0.0086 | 0.0926 | 1.31, P = 0.25 | 1.92 |
| ORIGIN excluded | 10 | 14.72, P = 0.14 | 32.0% | 0.0096 | 0.0981 | 1.24, P = 0.27 | 1.86 |
| Risk & Prevention excluded | 10 | 15.09, P = 0.13 | 33.7% | 0.0082 | 0.0903 | 1.25, P = 0.26 | 1.87 |
| ASCEND excluded | 10 | 14.40, P = 0.16 | 30.6% | 0.0067 | 0.0816 | 0.80, P = 0.37 | 1.49 |
| VITAL excluded | 10 | 15.94, P = 0.10 | 37.3% | 0.0094 | 0.0972 | 1.49, P = 0.22 | 2.10 |
| STRENGTH excluded | 10 | 12.81, P = 0.23 | 21.9% | 0.0045 | 0.0668 | 0.50, P = 0.48 | 1.28 |

**Supplemental Table 5.** Results of study risk of bias assessment using the Cochrane Risk of Bias 2 tool

| **Study** | Domain 1.  Randomization Process | Domain 2.  Deviations from intended interventions | Domain 3.  Missing outcome data | Domain 4.  Measurement of the outcome | Domain 5.  Selection of the reported result | Overall bias | Composite risk of bias (RoB) score |
| --- | --- | --- | --- | --- | --- | --- | --- |
| JELIS | Low | Some concerns | Low | Low | Low | Some concerns | 2 |
| Nosaka et al | Low | Some concerns | Low | Low | Low | Some concerns | 2 |
| REDUCE-IT | Low | Low | Low | Low | Low | Low | 1 |
| RESPECT-EPA | Low | High | Low | Low | Low | Some concerns | 2 |
| IEIS-4 | Low | Low | Low | Low | Some concerns | Some concerns | 2 |
| GISSI-P | Low | High | Low | Low | Low | High | 3 |
| OFAMI | Low | Low | Low | Low | Some concerns | Some concerns | 2 |
| Raitt et al. | Low | Low | Low | Low | Some concerns | Some concerns | 2 |
| DO IT | Low | Low | Low | Low | Some concerns | Some concerns | 2 |
| GISSI-HF | Low | Low | Low | Low | Low | Low | 1 |
| Alpha Omega | Low | Low | Low | Low | Low | Low | 1 |
| ORIGIN | Low | Low | Low | Low | Low | Low | 1 |
| Risk & Prevention | Low | Low | Low | Low | Low | Low | 1 |
| ASCEND | Low | Low | Low | Low | Low | Low | 1 |
| VITAL | Low | Low | Low | Low | Low | Low | 1 |
| STRENGTH | Low | Low | Low | Low | Low | Low | 1 |

**Supplemental Table 6.** Heterogeneity statistics for meta-analysis of reported cardiovascular mortality hazard ratios comparing EPA/DHA + statin to statin therapy.

| Pooled Analysis | DF | Q, P-value | I^2^ | Tau^2^ | Tau | Likelihood ratio test statistic, P-value | Likelihood ratio |
| --- | --- | --- | --- | --- | --- | --- | --- |
| EPA/DHA | 1 | 1,84, P = 0.18 | 45.5% | 0.0104 | 0.1021 | 0.23, P = 0.63 | 1.12 |

**Supplemental Table 7.** Heterogeneity statistics for meta-analysis of reported cardiovascular mortality hazard ratios comparing EPA/DHA to standard preventive therapy among primary CVD prevention cohorts.

| Pooled Analysis | DF | Q, P-value | I^2^ | Tau^2^ | Tau | Likelihood ratio test statistic, P-value | Likelihood ratio |
| --- | --- | --- | --- | --- | --- | --- | --- |
| Full model | 2 | 1.34, P = 0.51 | 0.0% | 0.0004 | 0.0202 | 0.002, P = 0.96 | 1.00 |
| EPA | 0 | - | - | - | - | - | - |
| EPA/DHA | 1 | 1.07, P = 0.30 | 6.1% | 0.0008 | 0.0275 | 0.002, P = 0.96 | 1.00 |

**Supplemental Table 8.** Heterogeneity statistics for meta-analysis of reported cardiovascular mortality hazard ratios comparing EPA/DHA to standard preventive therapy among secondary CVD prevention cohorts.

| Pooled Analysis | DF | Q, P-value | I^2^ | Tau^2^ | Tau | Likelihood ratio test statistic, P-value | Likelihood ratio |
| --- | --- | --- | --- | --- | --- | --- | --- |
| Full model | 7 | 14.29, P = 0.046 | 51.0% | 0.0364 | 0.1909 | 3.69, P = 0.055 | 6.32 |
| EPA | 2 | 2.73, P = 0.26 | 26.7% | <0.0001 | 0.0029 | 0.00, P = 1.00 | 1.00 |
| EPA – Nosaka et al. excluded | 1 | 0.33, P = 0.57 | 0.0% | <0.0001 | <0.0001 | 0.00, P = 1.00 | 1.00 |
| EPA/DHA | 4 | 10.24, P = 0.037 | 60.9% | 0.0428 | 0.2068 | 3.85, P = 0.0497 | 6.86 |

Supplemental References

1. Khan SU, Lone AN, Khan MS, et al. Effect of omega-3 fatty acids on cardiovascular outcomes: A systematic review and meta-analysis. *EClinicalMedicine*. Aug 2021;38:100997. doi:10.1016/j.eclinm.2021.100997

2. Miyauchi K, Iwata H, Nishizaki Y, et al. Randomized Trial for Evaluation in Secondary Prevention Efficacy of Combination Therapy-Statin and Eicosapentaenoic Acid (RESPECT-EPA). *Circulation*. Aug 6 2024;150(6):425-434. doi:10.1161/CIRCULATIONAHA.123.065520

3. Sheppard JP, Palatnic L, Lakshmanan S, et al. Effects of purified eicosapentaenoic acid (EPA) and docosahexaenoic acid (DHA) on cardiovascular endpoints: The current view from randomized clinical trials [CRD42023390587]. *PROSPERO: International prospective register of systematic reviews*. 1/20/2023 2023;

4. Nosaka K, Miyoshi T, Iwamoto M, et al. Early initiation of eicosapentaenoic acid and statin treatment is associated with better clinical outcomes than statin alone in patients with acute coronary syndromes: 1-year outcomes of a randomized controlled study. *Int J Cardiol*. Feb 1 2017;228:173-179. doi:10.1016/j.ijcard.2016.11.105

5. Sterne JAC, Savovic J, Page MJ, et al. RoB 2: a revised tool for assessing risk of bias in randomised trials. *BMJ*. Aug 28 2019;366:l4898. doi:10.1136/bmj.l4898

6. Schwarzer G. meta: An R package for meta-analysis. *R News: The Newsletter of the R Project*. 2007;7(3):40-45.

7. Viechtbauer W. Conducting meta-analyses in R with the metafor package. *Journal of Statistical Software*. 2010;36(3):1-48.

8. Lin L, Chu H. Quantifying publication bias in meta-analysis. *Biometrics*. Sep 2018;74(3):785-794. doi:10.1111/biom.12817

9. Buchan IE. Heterogeneity in Meta-analysis. Stats Direct: StatisticalHelp. <https://www.statsdirect.com/help/meta_analysis/heterogeneity.htm>

10. Higgins JP, Thompson SG, Deeks JJ, Altman DG. Measuring inconsistency in meta-analyses. *BMJ*. Sep 6 2003;327(7414):557-60. doi:10.1136/bmj.327.7414.557
